# Antioxidants Attenuate Heat Shock Induced Premature Senescence of Bovine Mesenchymal Stem Cells

**DOI:** 10.3390/ijms23105750

**Published:** 2022-05-20

**Authors:** Dana Nir, Ivana Ribarski-Chorev, Chen Shimoni, Carmit Strauss, Jan Frank, Sharon Schlesinger

**Affiliations:** 1 Department of Animal Sciences, The Robert H. Smith Faculty of Agriculture, Food, and Environment, The Hebrew University of Jerusalem, Rehovot 7610001, Israel; dana.nir2@mail.huji.ac.il (D.N.); ivana.ribarski-chorev@mail.huji.ac.il (I.R.-C.); chen.shimoni@mail.huji.ac.il (C.S.); carmit.feliks@mail.huji.ac.il (C.S.); 2 Department of Food Biofunctionality, Institute of Nutritional Sciences, University of Hohenheim, D-70599 Stuttgart, Germany; jan.frank@nutres.de

**Keywords:** antioxidants, senescence, heat shock, mesenchymal stem cells, melatonin, resveratrol, curcumin

## Abstract

Mesenchymal stem cells (MSC) have many roles that are important for the body’s proper functioning. When the MSC pool is damaged, it is often correlated with impaired development or health of the organism. MSC are known for their anti-inflammatory, immunomodulatory and trophic characteristics that play an important role in the physiological homeostasis of many tissues. Heat shock impairs MSC capacity by inducing the generation of reactive oxygen species and mitochondrial dysfunction, which, in turn, send the cells into a state of premature senescence. Here, we pre-exposed MSC to melatonin, resveratrol, or curcumin, which are natural antioxidative compounds, and tested the protective effects of these substances from oxidative stress and aging. Our data showed that pre-exposure of MSC to antioxidants decreased reactive oxygen species while mitochondrial damage remained high. Additionally, although the proliferation of the cells was slow, antioxidants protected the cells from premature senescence, and subsequent cytokine release was prevented. We conclude that while elevated temperatures directly cause mitochondrial damage, senescence is induced by elevated ROS levels. We suggest that heat shock alters cell and tissue homeostasis by several independent mechanisms; however, reducing tissue senescence will reduce damage and provide a pathway to overcome physiological challenges in animals.

## 1. Introduction

The frequency of heatwaves is increasing throughout the world, with potentially dire consequences for human wellbeing and farm animal health. In bovines, the frequency of many chronic inflammation-related diseases is elevated during a hot period [1,2], resulting in impaired animal welfare and economic losses to the dairy industry [3,4]. At the cellular level, heat stress increases the concentration of intracellular reactive oxygen species (ROS) in cells [5], which react with DNA, proteins, and other macromolecules, leading to an accumulation of mutations and misfolded proteins [6]. Moreover, increased ROS levels elicit stem cell depletion and functional defects in several tissues [7]. Adult stem cells are the longest living cell population in the body and are therefore vulnerable to stressful environmental conditions that might mitigate their function. However, the link between the physiological influences of summer heat stress on the cow and the effect of elevated temperature on stem cells remains a mystery.

Mesenchymal stromal cells (also called mesenchymal stem cells, MSC) are a heterogeneous group of non-hematopoietic multipotent stem cells belonging to the mesoderm layer. They are known for their multi-lineage differentiation ability into cell types such as adipocytes, osteoblasts, chondrocytes, and others [8,9] and for their anti-inflammatory, immunomodulatory, and trophic characteristics, which induce immune tolerance in diverse inflammatory conditions [10,11]. Physiological MSC stores are essential for the regeneration of many tissues in the body, including the uterus [12], where they are essential for the rehabilitation after each ovulation cycle and especially after birth [13]. Although the physiological role of umbilical cord MSC is not clear, MSC extracted from patients with pregnancy-related disorders showed higher oxidative stress and reduced viability of co-cultured trophoblast cells [14].

Another example is the decrease in the regenerative capacity of MSC cells with age. The “dormant” pool of cells disappears completely in old age as the MSC pool is damaged following exposure to harsh environmental conditions [15,16]. Taken together, MSC have many roles in the body that are important for its proper function, but when the MSC pool is damaged, it is often correlated with impaired development or health issues. This is consistent with both our and other previous findings of the high sensitivity of MSC to oxidative stress [17], which increases with heat stress [18].

Exposure of bovine umbilical cord MSC to heat shock (HS), that is, high temperatures of 40.5 °C or 42 °C, leads to mitochondrial damage and oxidative stress, which, in turn, results in critical changes in the proliferation, differentiation, and immunomodulatory phenotype [18]. The purpose of using these two temperatures was to model both the situation of a mild but steady increase in environmental or bodily temperature (40.5 °C, for 72 h) and that of a short exposure to extreme heat stress in the middle of a summer day exposed to the sun (42 °C, for 1 h). A short HS of 1 h at 42 °C was followed by a 71-h recovery at 37 °C to evaluate the long-term effects that could still be observed several passages later. Interestingly, both of the in vitro HS protocols significantly increased cellular aging, a phenomenon termed stress-induced premature senescence (SIPS). The senescence-associated secretory phenotype is characterized by soluble cytokines, growth factors, and enzymes [19], with a disruptive influence on the surrounding tissue homeostasis. Therefore, elevated temperatures can induce HS on tissue MSC, which will cause early aging of the stem cell pool and deliver inflammatory signals to the surrounding tissue. Hence, we hypothesize that protecting MSC from HS damage will eventually improve the amount, efficacy, and functionality of MSC and improve the performance and health of cows during the hot summer months.

In vitro, pre-conditioning of MSC with antioxidants can improve their resistance against stressors. For example, pre-conditioning of MSC with resveratrol [20], melatonin [21], or curcumin [22] prevented MSC death caused by H_2_O_2_. Therefore, these three natural antioxidants were chosen here for study.

Resveratrol is a phytochemical with many reported biological effects, including that of an antioxidant, at least in vitro [20]. Resveratrol effectively inhibits the generation of reactive oxygen and nitrogen species and scavenges excessive radicals in liver cells. It activates SIRT1 and thus inhibits entry into cellular senescence and preserves the ability of stem cells for self-renewal [23]. Its effect varies, and different concentrations have shown opposite effects [24], and cells in various passages were affected differentially [25].

Melatonin is a hormone secreted from the pineal gland in the brain, known for its role in controlling sleep and wakefulness in animals. Studies have shown that melatonin plays a key role in protecting MSC and can control cell fate [26]. Melatonin lowers ROS accumulation by activating antioxidant enzymes, which affect various molecular pathways in the cell, such as inflammation, proliferation, and apoptosis [27,28]. Reduced mitochondrial damage and ROS accumulation following melatonin treatment [29,30,31] lead to a reduced SIPS phenotype [29,32].

Curcumin is a phytochemical isolated from turmeric (*Curcuma longa*) with reported antioxidant, anti-inflammatory, antimicrobial, antiviral, and antifungal activities [33]. In vitro, curcumin is an efficient antioxidant, while inside cells, it induces the expression of endogenous antioxidant proteins via Nrf2 [33,34,35,36]. Curcumin reduces ROS accumulation and mitochondrial damage, prevents mitochondrial dysfunction and induces mitochondrial fusion [37], reduces apoptosis, and promotes MSC survival [22]. After some time inside the cell, it is metabolized to conjugates that are redox-silent and therefore have no direct antioxidative effect.

Our goal was to study the effects of these antioxidants on the function of bovine MSC after heat shock in vitro. We hypothesize that pre-exposing bovine MSC to antioxidants will protect them or prevent some of the damage caused to the cells at high temperatures.

## 2. Results

### 2.1. Optimizing Antioxidant Treatments on MSC

Melatonin, resveratrol, and curcumin are widely tested in the context of regenerative medicine and cell therapy. As antioxidants, they are predicted to improve stem cell fitness and resistance to stress. Since these substances give varied and sometimes even opposite effects when used in different concentrations and on different cell types, we first calibrated their use on bovine MSC.

**Figure 1:**
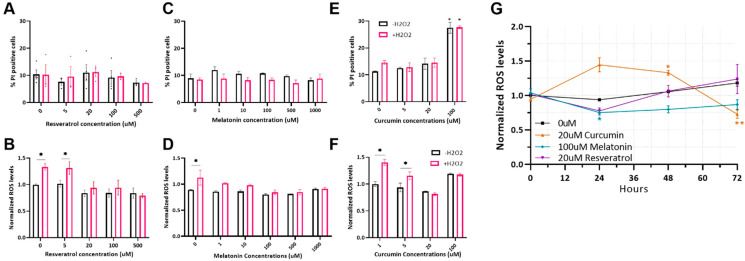
Calibration of antioxidant activity in MSC cells. (**A**) Percentage of dead cells by propidium iodide-staining and analysis by flow cytometry after incubation with resveratrol for 24 h at stated concentrations, with and without 500 µmol/L H_2_O_2_. (**B**) ROS levels in the cells as seen after staining with CellRox reagent and analysis by flow cytometry after incubation with resveratrol for 24 h at the indicated concentrations with and without 500 µmol/L H_2_O_2_. (**C**) Percentage of cells death after incubation with melatonin for 24 h were measured as in (**A**). (**D**) ROS levels in cells after incubation with melatonin for 24 h were measured as in (**B**). (**E**) Percentage of cells death after incubation with curcumin for 72 h were measured as in (**A**). (**F**) ROS levels in cells after incubation with curcumin for 72 h were measured as in (**B**). For (**A**–**F**), *n* = 3. Two-way ANOVA with Tukey test for multiple comparisons was used, different letters indicate significant differences (*p* < 0.05). (**G**) Analysis of ROS levels, after incubation with resveratrol, melatonin, and curcumin for 24, 48, and 72. Two-way ANOVA with Dunnett’s test for multiple comparisons was used for statistical analysis (* = *p* < 0.05, ** = *p* < 0.001), *n* = 6.

Resveratrol was incubated with bovine MSC for 24 h with a non-toxic effect at concentrations ranging from 5 to 100 µmol/L (Figure 1A). A dose-dependent decrease in cellular ROS in cells treated with H_2_O_2_ was observed at concentrations of 20–500 µmol/L (Figure 1B); thus, a 20 µmol/L concentration was selected for further study.

Melatonin addition for 24 h had no cytotoxic effects on the MSC at the concentrations tested (Figure 1C). A significant reduction in ROS levels in cells treated with H_2_O_2_ was seen starting from a concentration of 100 µmol/L (Figure 1D), which was the concentration selected for further use.

Curcumin is known to induce opposite effects under different conditions, depending on the time of treatment, concentration, and stem cell type [36,38]. Many studies have shown that longer incubation times can maximize the beneficial effects of this substance [36,39]. Therefore, we first examined the optimal time of curcumin treatment in culture and found that only a long incubation (>48 h) reduces ROS levels. Consequently, we incubated curcumin with the cells for 72 h. The 20 µmol/L concentration was non-toxic (Figure 1E) but reduced the amount of ROS in cells treated with H_2_O_2_ (Figure 1F) and was therefore chosen for further use.

Next, we examined the optimal duration of treatment—long enough for protection but before the substance breaks down and loses activity—for all antioxidants. It was found that the incubation time greatly affected the antioxidative activity. A 24 h incubation was found to be optimal for resveratrol and melatonin, while curcumin’s protective effect was observed only after 72 h (Figure 1G). No effect on cell viability was observed (data not shown).

### 2.2. Antioxidant Protection of MSC from Heat-Shock-Induced Oxidative Stress

**Figure 2:**
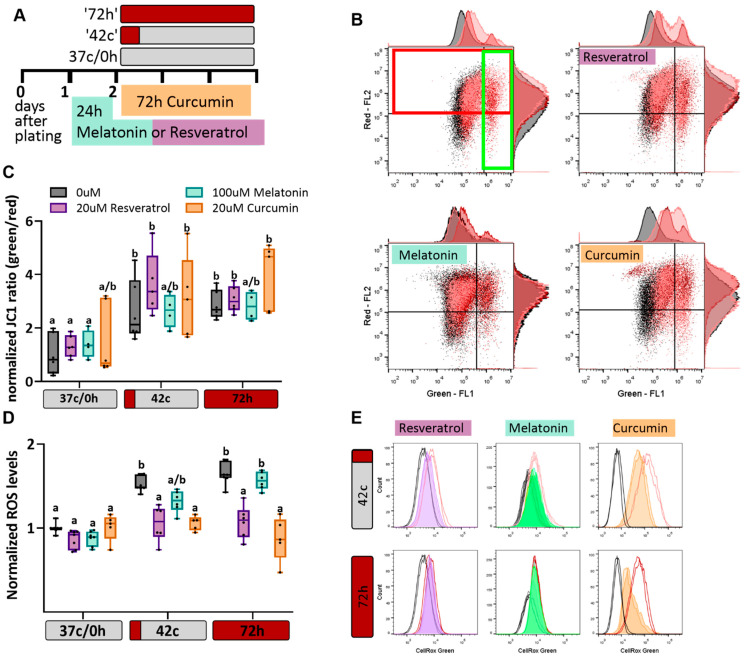
The effect of antioxidants on mitochondria and ROS. (**A**) Schematic illustration of the experimental design: bovine umbilical cord-MSC are plated and 24 h later treated with resveratrol or melatonin. On day 2 the cells are placed at 42 °C for 1 h and then allowed to recover at 37 °C for three days (42 c) or at 40.5 °C for 3 days (72 h). Curcumin is added on day 2 with the heat shock. (**B**) Membrane potential was measured using JC-1 dye and flow cytometry. Representative results are shown. Cells before and after heat shock treatment (black and red dots/histograms, respectively. Bright red indicates 42 °C HS and dark red histogram indicates 72 h HS) are shown with or without antioxidant addition to the growth media. Percent of cells showing mitochondrial depolarization is increased after short and long heat shock as compared to the untreated cells. (**C**) Summary of six biological repeats. Two-way ANOVA with FDR rates calculated using two-stage linear step-up procedure of Benjamini, Krieger and Yekutieli was used for statistical analysis, different letters indicate significant differences (*p* < 0.05), where two letters appear-significant difference was observed from neither sample. (**D**) Flow cytometry results of CellROX staining for cellular ROS levels after heat shock, treated with the three antioxidants. Data presented are a mean of green fluorescence as compared to 37 °C, 0 h, *n* = 6. Two-way ANOVA with FDR rates calculated using two-stage linear step-up procedure of Benjamini, Krieger and Yekutieli was used for statistical analysis, different letters indicate significant differences (*p* < 0.05), as in (**C**). (**E**) An example from the data summarized in (**D**).

MSC at early passages (P3–P5, see Supplementary Figure S1 for characterization) were moved from 37 °C (Figure 2A, ‘37 c/0 h’) to either 42 °C for 1 h heat shock followed by a three-day recovery back to 37 °C (Figure 2A, ‘42 °C’) or 40.5 °C for 72 h (Figure 2A, ‘72 h’). Mitochondrial membrane potential (MMP) was quantified based on the percentage of green cells (low MMP) vs. red cells (high MMP; Figure 2B). Both heat shock protocols resulted in mitochondrial damage in MSC after three days (Figure 2B,C, grey boxes). Although the heat shock lowered the potential of the membranes, the antioxidant had no protective effect on the cells (Figure 2B,C). Nevertheless, the addition of resveratrol, melatonin, or curcumin significantly decreased the amount of ROS in the cells exposed to the short heat shock protocol. After 72 h of heat shock, melatonin was no longer effective, while resveratrol and curcumin still protected the cells (Figure 2D,E). This suggests that the mitochondrial damage seen after heat shock is not the result of oxidative stress and cannot be recovered by treatment with antioxidants. Conversely, HS-induced oxidative stress was relieved following the treatment, showing the protocol’s effectiveness in these conditions.

### 2.3. Antioxidants Have No Effect on Heat Shock-Induced Reduction in Cell Proliferation

**Figure 3:**
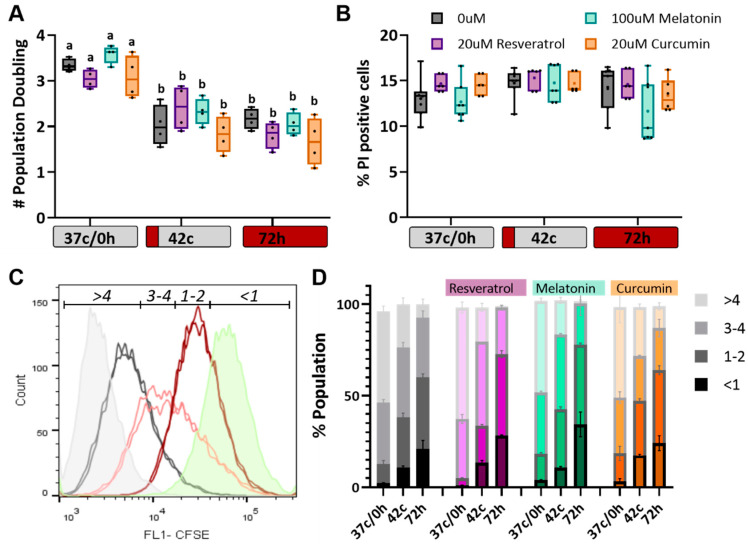
The effect of resveratrol, curcumin, and melatonin on the viability and proliferation of MSC. (**A**) Population doubling time was calculated after 5 days in culture. *n* = 4. Two-way ANOVA with multiple comparisons test FDR rates calculated using two-stage linear step-up procedure of Benjamini, Krieger and Yekutieli was used for statistical analysis, different letters indicate significant differences (*p* < 0.05). (**B**) Propidium iodide staining on unfixed cells was performed to quantify the number of dead cells in the population following heat shock and/or antioxidant treatments. *n* = 6. No significant difference was found. (**C**) CFSE staining in cells before and after short or long heat shock treatment (black and pink and red, respectively) is shown. Undivided cells are used as a positive control (green) and unstained cells as a negative (black). The gates used for further analysis are shown. (**D**) Mean percentage of cells in each state are shown, *n* = 6. No significant difference detected as measured by Two-way ANOVA.

We previously reported that following heat shock, both short and long, MSC does not die but slow their proliferation rate significantly and enter a state of senescence. To study the effect of antioxidant treatment on these phenomena, population doublings were counted at the end of the 72-h treatments (Figure 3A), and propidium iodide staining was used to measure the percentage of dead cells (Figure 3B), but no significant protection of antioxidant treatments was observed. Therefore, we turned to quantify the number of cell divisions by using a CFSE dye. This dye can penetrate live cells, but then the staining fades with each cell division linearly. The dye was added on day 2 at the beginning of the heat shock treatment, and analysis was performed three days later by flow cytometry. The populations were divided into four states—<1 division (highest CFSE fluorescence), 1–2, 3–4, and >4 cell divisions (when fluorescence is below detection, see example in Figure 3C). Positive control cells were stained just before the analysis (no cell division could occur yet; green histogram), and unstained cells were used as negative control (gray histogram). The results show that heat shock delayed proliferation and that the antioxidants did not affect this slowdown (Figure 3D). However, many studies suggest that antioxidants can induce G2/M phase arrest in some cells [40]. Accordingly, we examined the ability of the antioxidant treatments to protect MSC from premature senescence.

### 2.4. Antioxidants Protection of MSC from Heat-Shock Induced Premature Senescence

**Figure 4:**
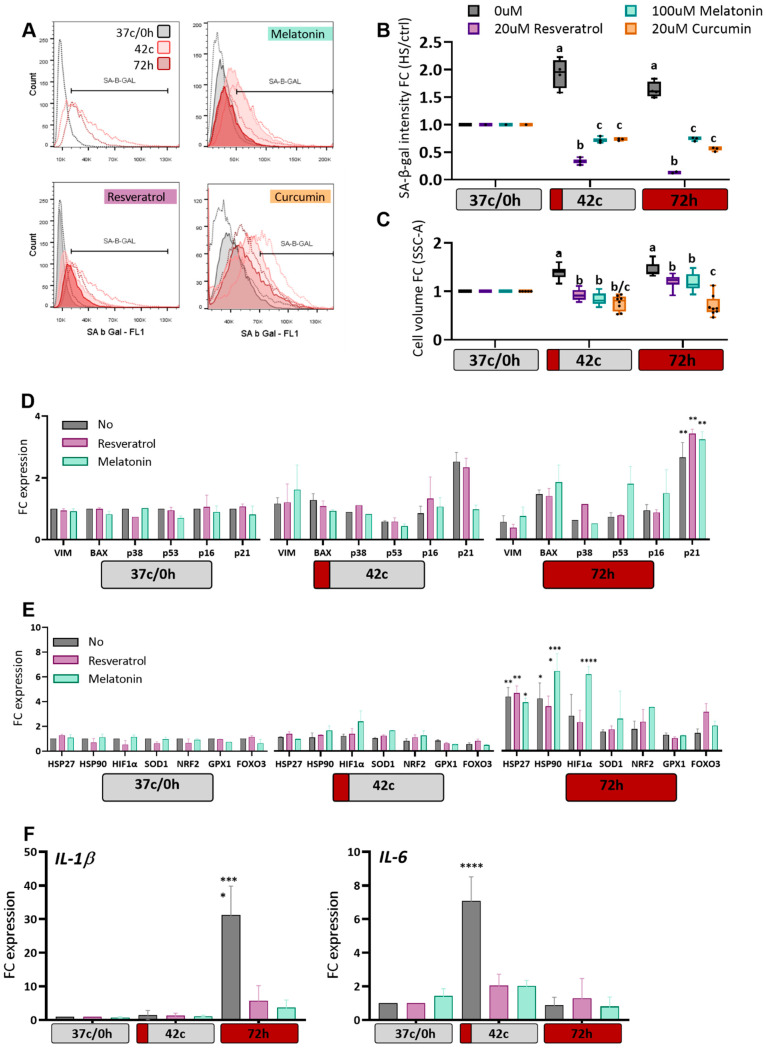
Attenuation of heat shock-induced senescence by antioxidants. (**A**) SA-β-gal staining results from one representative experiment are shown as an overlay of cells without (black) or with short (pink) or long (red) heat shock. (**B**) flow cytometry data of mean green fluorescence intensity, ± SEM for *n* = 6. All values were normalized to 37 c/72 h group, which was set at 1.0 as the control. (**C**) Side scatter geometric mean (e.g., volume of the cells) of cells following different treatments are shown. Flow cytometry data of mean green fluorescence intensity, ± SEM for *n* = 6. All values were normalized to 37 c/72 h group. (**D**) Cell markers-, cell cycle-, and apoptosis-related genes expression evaluated using RT-qPCR and normalized to housekeeping control genes (Proteasome Subunit Beta2-PSMB2 and Ribosomal protein S9-RPS9). (**E**) Same, but for heat shock proteins and stress responsive genes. (**F**) SAPS factors IL-1b and IL-6 expression. For all data *n* = 6, Two-way ANOVA with Tukey test for multiple comparisons was used for (**B**,**C**), different letters indicate significant differences (*p* < 0.05). Two-way ANOVA with Dunnett’s test for multiple comparisons was used in (**D**–**F**), significant differences indicated by * *p* < 0.05, ** *p* < 0.01, and **** *p* < 0.0001.

A cell cycle halt can indicate the entry of cells into a state of senescence or cellular aging. We examined whether the reduced ROS levels following antioxidant treatments would lead to a decrease in the senescence phenotype, as measured by SA-β-Gal (Senescence-associated beta-galactosidase) staining, which is a marker for detecting cellular senescence [41]. Increased expression of beta-galactosidase was observed in cells that experienced heat shock (Figure 4A, upper left). This increase was reduced and even eliminated following treatments with all three antioxidants (Figure 4A,B). The increase in cell volume, another marker of senescence, was also somewhat rescued by the addition of the antioxidant (Figure 4C and Figure S2). To further validate this effect, we examined the expression changes of several stress response and cell cycle genes. As expected, following HS, a significant increase was observed in the expression of p21 (CDKN1a) (Figure 4D), a cell cycle inhibitor and major regulator of the senescence program [42]. This upregulation was prevented in the melatonin-treated population after the 42 °C HS protocol but not after the 72-h HS. Other cell cycle- or apoptosis-related genes, such as p53, BAX, or p38 (MAPK), were not upregulated, nor was the common senescence marker p16 (CDKN2A), in agreement with previous findings [18,43]. Stress-response genes such as heat shock proteins and hypoxia-inducible factor α were upregulated following the 72 h HS protocol; however, this was not prevented by the antioxidant treatment (Figure 4E). Last, we examined two typical senescence-associated secretory phenotype factors and observed that while IL-1b, which is known as an early responder [44], is upregulated in the 72 h HS (i.e., immediately after heat shock), the late IL-6 gene is upregulated after recovery from the 42 °C HS (Figure 4F). Unfortunately, we were unable to extract good quality RNA from the curcumin-treated cells, but nonetheless, the treatment with both melatonin and resveratrol prevented the senescence-associated secretory phenotype. Thus, we conclude that treatment with melatonin or resveratrol can protect MSC from some heat shock-induced cellular damage.

## 3. Discussion

Despite thermal adaptation mechanisms, heat stress breaches animal body homeostasis, thereby depressing their production and productivity. Here, we wished to examine the effects of acute and chronic heat stress on the mesenchymal stem cell pool, which can only be isolated based on their ability to proliferate and differentiate in vitro. The HS treatment, performed with a control on the same MSC line, allowed us to compare the cellular response without the effect of variables such as the genetic background or behavior that exist when comparing different animals.

At the cellular level, we can verify the cells examined for various stem cell markers and functions before performing the experiment and thus focus our attention on these specific cells. The 42 °C for 1 h treatment was chosen since previous reports [45] used these conditions to study the immediate effect on cells, but none examined the long-term lasting effect of a short HS after three days. This is physiologically relevant since cells are rarely exposed to long severe heat stress but they might experience short severe heat stress with an extremely high-temperature humidity index (THI), especially if the cows are pregnant, lying down, and not cooled in the mid-day. Therefore, in the ‘short’ HS protocol, we tried to simulate the long-term effect of extremely hot days, and in the ‘long’ HS, we simulated the effect of chronic exposure to the average high THI typical of hot climates.

Mesenchymal stem cells exhibit various cellular damages following heat shock. The cells enter oxidative stress due to the accumulation of ROS molecules in the cells, the ratio of dysfunctional mitochondria increases, the rate of proliferation decreases, and an increase is seen in the number of cells entering SIPS [18]. Interestingly, these effects are observed as long as three days after the 1 h HS was applied, suggesting a long-term drop in the state of the stem cell pool in heat-stressed animals and possibly explaining the chronic reaction of animals to thermal stress [46]. Here, we examined the efficiency of antioxidant treatments in protecting MSC from the above damage. Pre-treatment with melatonin and resveratrol was tested, as well as long treatment with curcumin. These three substances were suggested as an effective treatment for stress, acting by various metabolic and cellular pathways [47]. Resveratrol pre-treatment effectively restricted the increase in ROS after a short but not long heat shock and reduced the number of cells entering senescence. Melatonin presented a similar effect, although it was slightly less effective in controlling ROS accumulation. Exposing cells to curcumin for 72 h, simultaneously with a long or short heat shock, prevented ROS accumulation and entry into senescence. No effect on viability, proliferation, or mitochondrial function was observed.

Heat shock causes oxidative stress in cells, a violation of the oxidative balance in the cell [5]. This condition exists due to either the accumulation of ROS or a lack of natural antioxidants to control the basal ROS levels [48]. Therefore, an antioxidant treatment, as expected, was able to moderate and prevent the accumulation of ROS. Studies have shown that melatonin, curcumin, and resveratrol act either directly, by inactivating reactive species, and/or indirectly, through induction of the expression of antioxidant enzymes, such as SOD1, by activating transcription factors such as Nrf2 [26,49,50]. In our experiments, no effects of antioxidant treatments on the expression of these genes were observed, in agreement with [51], thus suggesting a different route of action.

Oxidative stress can result in impaired mitochondrial function [52,53]. However, the mitochondrial function also affects the cellular ROS balance [54], and thus mitochondrial damage can lead to apoptosis, disrupted immune system signals, and subsequently damage and disease to the organism [50]. Short and long heat shocks reduced both mitochondrial functions (i.e., a high proportion of cells with low membrane potential vs. normal mitochondria) and induced oxidative stress (i.e., high ROS levels) in MSC. While the antioxidant treatments were able to reduce the ROS levels, the damage to the mitochondria remained, suggesting that the heat shock had a destructive effect on these organelles. Nonetheless, our data indicate that the high ROS levels might be partially the outcome of mitochondrial damage. This finding might explain the fact that antioxidants, while reducing ROS in bovine oocytes, do not improve the fertility of oocytes exposed to heat shock [55].

Heat stress and oxidative stress cause SIPS in MSC [42,56]. Reducing oxidative stress by the antioxidant treatment was followed by reduced ROS and lower levels of SA-β Gal. Senescence is a state of cell cycle arrest, but in our experiments, the antioxidants did not restore proliferation in the heat-shocked cells. This might be due to the independent effect that the antioxidants have on the cell cycle. For example, resveratrol induces cell cycle arrest in cancer cells [57], and melatonin and curcumin inhibit cell proliferation [58,59] as well. Therefore, the antioxidants release the MSC from the SIPS but induce parallel proliferation to slow down in a distinct pathway. This hypothesis is supported by the unchanged expression levels of p53. Senescent cells have several additional parameters that characterize them, such as flat and swollen morphology, increased SA-β Gal activity [41], and the development of a pro-inflammatory secretory phenotype [60,61]. These senescence markers were not observed in the antioxidant-treated cells, supporting the SA-β Gal test and the attenuation of SIPS.

Last, we established that heat stress triggers a complex program of gene expression and cellular adaptive responses, which can be partially prevented by the addition of antioxidants to the cells before the HS. Despite the effect that the antioxidant treatments had on the SA-β Gal marker, only the melatonin pre-treatment inhibited the upregulation of p21 expression following the 42 °C HS. This might be due to the timing in which we extracted the RNA, four days after antioxidant treatment and three days after HS, thus probably missing immediate transcriptional changes. In the 72-h HS protocol, we found upregulation of stress response genes such as heat shock proteins and hypoxia-inducible factor, α, as expected. Not surprisingly, the antioxidant treatments had no effect on these genes. More surprising is the fact that the antioxidant pre-treatment did not seem to upregulate the endogenous antioxidant genes (NRF2, SOD1, etc.), contrary to previous findings [26,49,50]. A possible explanation might again be due to the time between the antioxidant treatment and the transcriptional analysis. This explanation is supported by the fact that P53 and P38, key genes in major biological processes and pathways enriched in response to thermal stress, were also not upregulated three days after the HS protocol initiation. Thus, the antioxidant effect is maintained even after the transcriptional reaction to HS is toned down.

Interestingly, although in many aspects, the cells do not seem affected by the antioxidant treatment, in other aspects we see significant differences, especially stress-induced premature senescence (SIPS) and senescence-associated secretory phenotype were avoided. Recent studies suggest that the accumulation of senescent cells in the tissue might result in widespread tissue damage due to the senescence-associated secretory phenotype effect [60,62,63,64]. This phenotype can exhaust the pool of immune cells in the body, which, in turn, leads to the failure of the immune system to evacuate the senescent cells and results in a positive feedback loop that gives rise to massive tissue damage or tumorigenic transformation [19]. Clearing senescent cells from mice has shown improvement in tissue function and reduced damage, suggesting that senescent cells affect both aging and lifespan [65,66].

The detrimental effects of heat stress on animal welfare will become more of an issue if the earth’s climate continues to warm as predicted. Changes in extreme temperatures will accompany continued global warming, thus raising the question of how animals will adapt to extreme temperature events. The harmful effects of heat stress are the result of either the hyperthermia associated with heat stress or the physiological adjustments made by the heat-stressed animal to regulate body temperature [55]. Our results suggest that antioxidants availability in the cells prior to the thermal stress induction can protect the cells from one harmful effect of HS, the premature aging of cells and tissues. This effect was suggested to be responsible for the high rates of inflammation and the poor health of animals in the hot season [60]. Antioxidant supplementation during the hot summer months may potentially be used as a novel treatment approach that is both affordable and efficient. More studies are needed to determine the bioactivity of these treatments in vivo.

## 4. Materials and Methods

### 4.1. Cell Culture

Bovine mesenchymal stromal cells (MSC), as we have previously reported [18], were isolated, cultured, and characterized based on generally accepted criteria [67,68] (Supplementary Figure S1). Briefly, 3 different umbilical cords were obtained from Israeli Holstein dairy cows immediately after birth. In total, 2 newborns were males and 1 female. In the lab, the umbilical cord was digested and plated as previously described in [69]. Cells were plated in a low-glucose Dulbecco modified eagle medium (Gibco, Carlsbad, CA, USA) containing 10% fetal bovine serum (Gibco) and a penicillin–streptomycin mixture (3%), expanded, and cryopreserved in fetal bovine serum containing 10% DMSO at passages 2–3. MSCs were examined and verified as in [18] before subsequent heat shock experiments. All the experiments described were performed on cells in passages 3–5.

### 4.2. Induction of Heat-Shock

For induction of heat shock, cells were divided into the following three groups: Control cells (37 c/0 h) were incubated at 37 °C for 72 h. Short HS cells (‘42 °C’) were exposed to 42 °C for 1 h and later moved to recovery at 37 °C for 71 h. Long HS cells (’72 h’) were exposed to 40.5 °C for 72 h. These conditions were chosen after other temperatures (39 °C) and time points (24, 48 h) were examined and the results indicated that cell damage was most evident in these treatments [18]. Similar results were also found in other studies [18,42,70]. It should be noted that the natural body temperature of cattle is 38.7 °C. Despite thermoregulation, rectal temperatures above 40 °C were measured during the summer months [55]. All cells were plated at 10^3^–10^4^/cm^2^ density (depending on the treatment protocol) two days before and taken for analysis immediately after the treatment protocol.

### 4.3. Antioxidant Calibration

The substances used were native resveratrol extract (30% trans-resveratrol, dissolved in ethanol, kindly donated by co-author Jan Frank), melatonin (M5250, SIGMA; dissolved in ethanol), and native curcumin (95%, dissolved in ethanol, donated by Jan Frank). The 10^4^/cm^2^ cells were plated in six-well plates and 24 h later antioxidants in the stated concentrations were added to the media. In total, 500 µmol/L H_2_O_2_ were added for the last 1 h at 37 °C to induce oxidative stress, where noted (+H_2_O_2_). Cells’ viability and ROS levels were examined after the stated times. Experimental protocol was set to the minimal concentration to eliminate excessive oxidative stress effect of the H_2_O_2_, which did not induce significant death in the culture as measured by propidium iodide (PI) staining.

### 4.4. Antioxidant Treatments

Twenty-four hours after plating, the respective antioxidant was added to culture media at the following given concentration: 20 µM resveratrol or 100 µM melatonin were added to the cells’ culture media for 24 h, washed, and the culture medium was replaced with fresh media. Thereafter, HS protocol was initiated. In total 20 µM curcumin was added to the media for 72 h, i.e., throughout the entire time of HS exposure.

### 4.5. Cell Death Quantification Using Propidium Iodide (PI)

For quantification of cell death in culture, cells were harvested by trypsinization, washed with PBS, and re-suspended in 10 µg/mL PI for 5 min on ice. Percentage of live/dead cells was determined within 1 h after staining by a CytoFLEX flow-cytometer (Beckman Coulter, Indianapolis, IN, USA). For each sample, 10,000 events were collected. At least three biological repeats were used for each treatment. Analysis of the samples was performed using Flow-Jo software.

### 4.6. Quantification of Reactive Oxygen Species

We used a CellROX Green Reagent (Invitrogen, Carlsbad, CA, USA), 0.5 µmol/L dye in PBS with 2% FBS for 1 h. As a positive control, cells were exposed for 1 h to 500 µmol/L H_2_O_2_ at 37 °C to induce oxidative stress. Cell cultures at 80–90% confluence were detached and separated using 0.05% trypsin–EDTA (T/E) solution, collected and measured using the CytoFLEX flow-cytometer (Beckman Coulter). For each sample, 10,000 events were collected. Analysis of the samples was performed using Flow-Jo software. At least three biological repeats were used for each treatment.

### 4.7. Mitochondrial Membrane Potential Measurement

We used a JC-1 assay (ENZO life sciences international, Farmingdale, NY, USA) as previously described [71]. Mitochondrial membrane potential was assessed based on the fluorescence emitted and classified into the following two main colors: red—high potential, and green—low potential. Three biological repeats were prepared for each treatment and a 1 h pre-staining incubation with 500 µmol/L H_2_O_2_ was prepared as a positive control. For more details, see [18].

### 4.8. Cell Proliferation Assay

In order to check the proliferation rate of the cells before the HS treatments, cells were washed with PBS and incubated for 20 min in PBS containing 0.5 µmol/L CellTrace CFSE Cell Proliferation Kit (Invitrogen), then washed with media and moved to HS treatments. After 72 h, cell fluorescence was measured. Positive control cells were stained on the day of analysis. Three biological repeats were used for each treatment.

### 4.9. Population Doubling (PD) Calculation

Following treatments, cells were trypsinized and counted. PD was calculated using the formula PDt = (days × log(2))/((log(N) − log(N0)). Where N is the cell number at the end and N0 the cell number at culture initiation.

### 4.10. Senescence-Associated β-Galactosidase Marker Assay

This was carried out using a Senescence Assay Kit (Abcam, Cambridge, UK) and measured by flow cytometry. HS treatment cells were incubated in warm media containing 0.15 µL dye, then washed twice in wash buffer and taken to be read by flow cytometry. Nine biological repeats were used for each treatment (from three different MSC sources).

### 4.11. RNA Extraction, Reverse Transcription (RT), and Quantitative PCR (RT-qPCR)

RNA extraction from cells was carried out as previously described [18] using PureLink RNA Midikit (Invitrogen). Unfortunately, we were unable to extract good quality RNA from the curcumin-treated cells, and therefore results are being shown for the other two antioxidant-treated cells only. RNA was then reverse-transcribed into cDNA using High-Capacity cDNA Reverse transcription kit (Applied Biosystems, Waltham, MA, USA). Real-Time PCR reactions were performed using Fast SYBR Green Master Mix (Applied Biosystems) in an ABI Step-One Plus Real-Time PCR system. To ensure validity, each sample was tested in triplicate (technical replicates). The relative mRNA fold change was calculated with the ΔΔct method [72], and the levels were normalized against those for bovine PSMB2 and RPS9. To maximize overlap between separate running plates, the “Create study” method was used in the StepOne Plus software, which enabled us to discard technical variations derived from different runs. All primers used were tested and found agreeable with standard curve evaluations and are listed in Table 1.

### 4.12. Statistical Analysis

All experiments were repeated three times on MSC extracted from the same fetus (male) umbilical cord in order to avoid batch effects. Key experiments (ROS levels, SA-b-Gal, and gene expression) were repeated with two other umbilical cord MSCs taken from female and male calves. *n* = 3 means that the HS protocol was performed three times on the same MSC, *n* = 6 means three biological repeats were performed on each of two different MSC, *n* = 9 means three biological repeats were performed on each of three different MSC).

For all experiments, data were presented as the mean ± SEM, where *n* denotes the number of repeats. Unless stated otherwise in the respective figure captions, statistical significance of the data was assessed using two-way ANOVA. Different letters indicate significant differences (*p* < 0.05) or as follows: * *p* < 0.05, ** *p* < 0.01, and **** *p* < 0.0001. Statistical analyses were done using GraphPad Prism (La Jolla, CA, USA). 

## 5. Conclusions

This study revealed that pre-treatment of mesenchymal stem cells with the antioxidants resveratrol, curcumin, and melatonin could protect MSC from oxidative stress-induced senescence.

## Figures and Tables

**Table 1:** Primers used for RT-qPCR.

Name	Target Gene	Sequence	Foreword/ Reverse
RPS9	Ribosomal protein S9	GAAGCTGATCGGCGAGTATG	Forward
RPS9	Ribosomal protein S9	GATCTTGGCCAGGGTGAAT	Reverse
PSMB2	Proteasome subunit beta 2	GATGCGAAATGGTTATGAACTG	Forward
PSMB2	Proteasome subunit beta 2	AGGTTTCGGCGAGTGAAAT	Reverse
HIF1	Hypoxia-inducible factor 1	ATTTTGGCAGCAATGACACA	Forward
HIF1	Hypoxia-inducible factor 1	CCAAATTTATATTCTGCAATTTCTCA	Reverse
NRF2	Nuclear factor erythroid 2	CCTCAAAGCACCGTCCTCAG	Forward
NRF2	Nuclear factor erythroid 2	CAATCAAATCCATGTCCTGCTGG	Reverse
FOXO3	Forkhead box protein O3	ACAAACGGCTCACTCTGTCC	Forward
FOXO3	Forkhead box protein O3	GTGCCGGATAGAGTTCTTCCA	Reverse
GPX1	Glutathione Peroxidase 1	CAACGGTGCGGGACTACA	Forward
GPX1	Glutathione Peroxidase 1	CCTCGTTCTTGGCGTTTTCC	Reverse
SOD1	Superoxide dismutase 1	AAGGGAGATACAGTCGTGGT	Forward
SOD1	Superoxide dismutase 1	CAAACTGATGGACGTGGAATC	Reverse
PRDX1	Peroxiredoxin 1	GTCACCTGGCATGGATCAAC	Forward
PRDX1	Peroxiredoxin	GACCCCATAGTCCTGAGCAA	Reverse
HSP90	Heat shock protein 90	GAGCAGTATGCCTGGGAGTC	Forward
HSP90	Heat shock protein 90	CCATTGGTTCTCCTGTGTCA	Reverse
HSP27	Heat shock protein 27	CCCTGGACGTCAACCACTT	Forward
HSP27	Heat shock protein 27	CTCGTGCTTGCCAGTGATCT	Reverse
BAX	Bcl-2-associated X	GCTTCAGGGTTTCATCCAGGA	Forward
BAX	Bcl-2-associated X	TCAGACACTCGCTCAGCTTC	Reverse
P38	Mitogen-activated protein kinase 14 (MAPK14)	TTCCAAGGGCTACACCAAGT	Forward
P38	Mitogen-activated protein kinase 14 (MAPK14)	TGGTTCAGCTGGTCAAGGTA	Reverse
P16	CDKN2A	CCCTCGTGCTGATGGCTAGT	Forward
P16	CDKN2A	CCCATCATCATCACCTGGTCTA	Reverse
P53	Tumor protein p53	ACTCTTCAGATCCGTGGGTTTA	Forward
P53	Tumor protein p53	CCATCCAGAGCATCCTTCAG	Reverse
P21	CDKN1A	CGCCAGCTGAGGTGTGAG	Forward
P21	CDKN1A	ATGGCACCTGTGGCTCTTCT	Reverse
VIM	Vimentin	GATGGACAGGTTATCAACGAAACT	Forward
VIM	Vimentin	TCCTTCTTGCTGGTAGTATTTTGC	Reverse
IL-1β	Interleukin 1 beta	AGCATCCTTTCATTCATCTTTGAAG	Forward
IL-1β	Interleukin 1 beta	GGGTGCGTCACACAGAAACTC	Reverse
IL-6	Interleukin 6	GGGCTCCCATGATTGTGGTA	Forward
IL-6	Interleukin 6	GTGTGCCCAGTGGACAGGTT	Reverse

## Data Availability

The data presented in this study are available on request from the corresponding author.

## Supplementary Materials

The following supporting information can be downloaded at: https://www.mdpi.com/article/10.3390/ijms23105750/s1.
