# Supplementary material for: Antioxidants Attenuate Heat Shock Induced Premature Senescence of Bovine Mesenchymal Stem Cells
Source: Int J Mol Sci. 2022 May 20;23(10):5750. doi: 10.3390/ijms23105750 (PMC9147428; doi:10.3390/ijms23105750)
Supplement: Supplementary file 1 [file ijms-23-05750-s001.zip › ijms-1703796-supplementary.pdf]

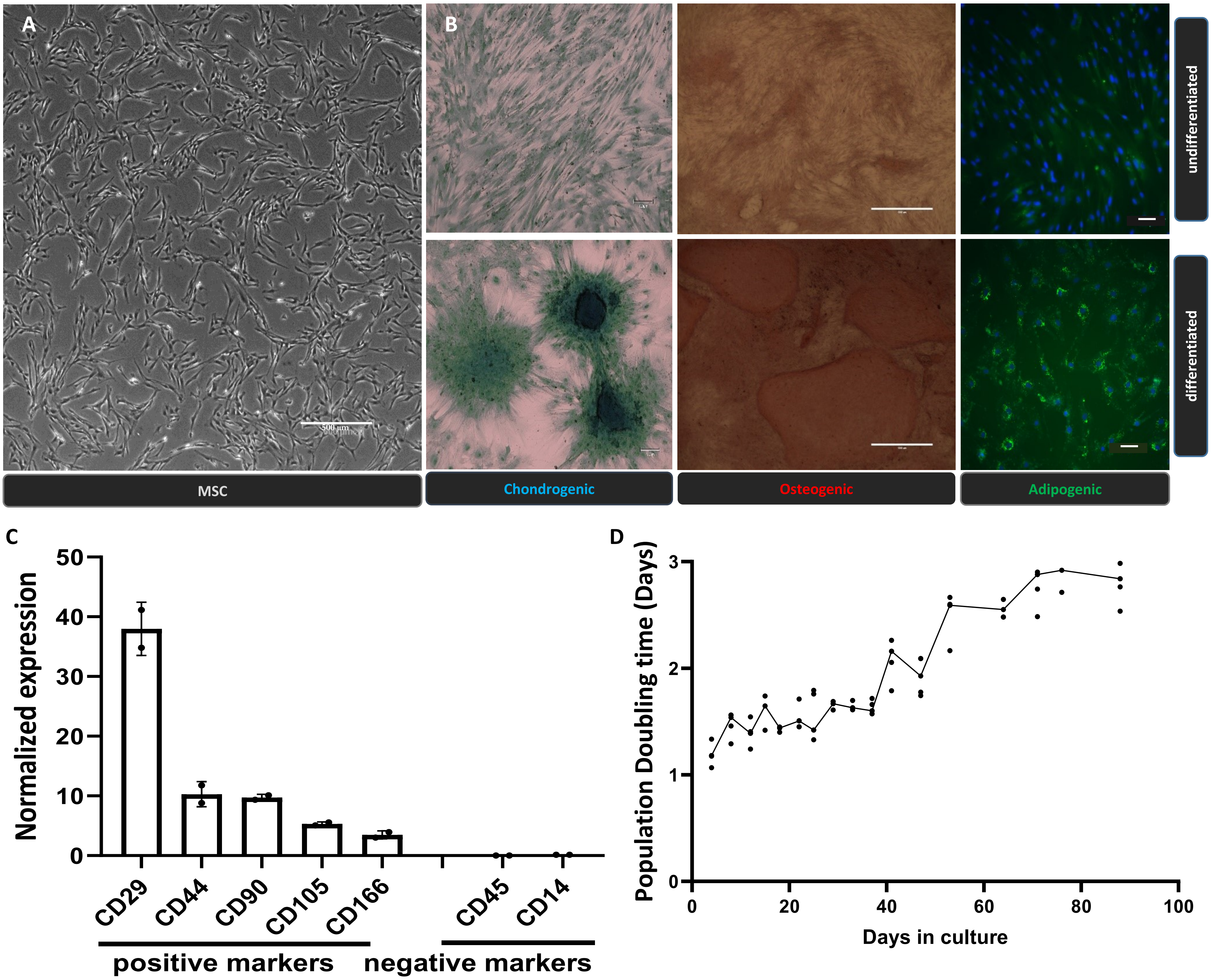

Figure S1: Characterization of MSC batch: (A) MSC at P2 prior do differentiation (Bright field, scale bar 500µm). (B) MSC differentiate to chondrocytes (Alcian blue staining), osteoblasts (Alizarin red staining, ImajeJ color adjustment mi, -14, max 240) and adipocytes (BODIPY™ 493/503 and DAPI staining). Chondrocyte and osteoblast staining was observed using EVOS® FL Auto microscope scale bar 100µm. Adipocyte staining was observed using Fluorescent microscope scale bar 50µm. (C) Expression of positive and negative MSC markers. (D) Population doubling time, 3-4 biological replicas at each time point. the line represent the median.

Figure S2: Cells after 42c or 72h HS treatments get enlarged and flattened, a typical aged / senescence morphology of MSCs. Bright field captures using Nikon Fluorescent microscope, scale bar 50µm. Pre-treatment with resveratrol, melatonin or curcumin can slightly alleviate the inflated morphology.

Figure S2

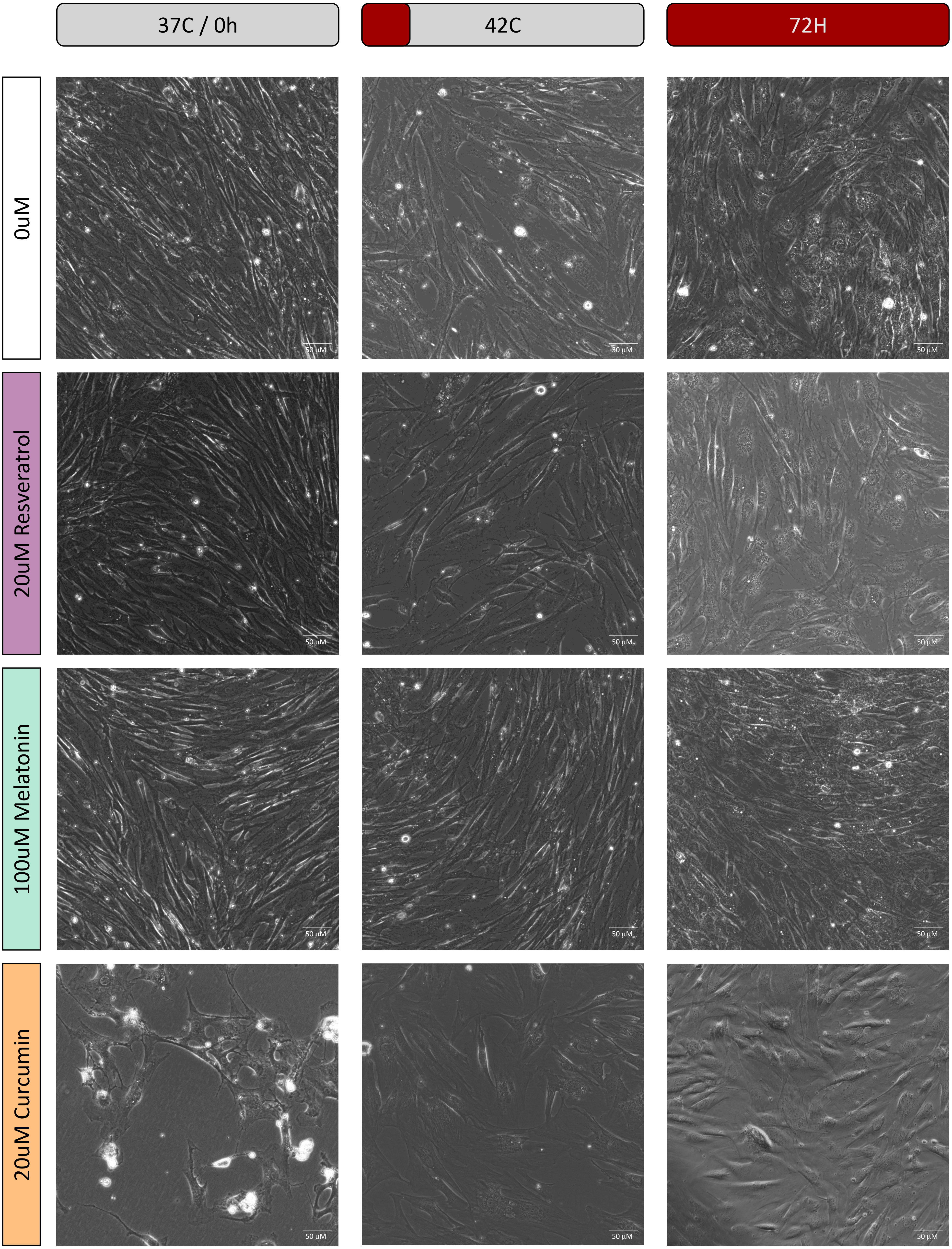

37C / 0h
42C
72H
0uM
20uM Resveratrol
100uM Melatonin
20uM Curcumin
50 µM
